# Two‐Stage Tumor Resection for Locally Advanced Renal Cell Carcinoma With Level IV Tumor Thrombus and Pulmonary Embolism: A Case Report

**DOI:** 10.1002/iju5.70045

**Published:** 2025-06-16

**Authors:** Takanari Kambe, Kei Mizuno, Yuki Teramoto, Takayuki Sumiyoshi, Yuki Kita, Kimihiko Masui, Takayuki Goto, Shusuke Akamatsu, Ryoichi Saito, Takashi Kobayashi

**Affiliations:** ^1^ Department of Urology Kyoto University Hospital Kyoto Japan; ^2^ Department of Diagnostic Pathology Kyoto University Hospital Kyoto Japan

**Keywords:** inferior vena cava thrombus, neoadjuvant therapy, pulmonary embolism, renal cell carcinoma, two‐stage surgery

## Abstract

**Introduction:**

We report a case of renal cell carcinoma with an inferior vena cava tumor thrombus extending into the right atrium and pulmonary embolism, treated using a staged surgical approach.

**Case Presentation:**

A man in his 60s was diagnosed with left clear cell renal cell carcinoma with a tumor thrombus extending to the right atrium, posing a risk of sudden death. Given the high perioperative risk, complete resection in a single session was infeasible. Preoperative administration of a tyrosine kinase inhibitor showed limited effectiveness, and the patient developed pulmonary embolism. An initial thoracotomy was performed to urgently remove the pulmonary artery and right atrial thrombus, along with as much infra‐diaphragmatic thrombus as feasible. This was followed by open radical nephrectomy and abdominal inferior vena cava thrombectomy.

**Conclusion:**

The staged approach enabled curative nephrectomy despite the presence of tumor thrombus and pulmonary embolism.

**Summary**

- Renal cell carcinoma with an inferior vena cava tumor thrombus and pulmonary embolism can severely affect the patient's condition, complicating curative surgery.
- This report highlights the successful use of staged surgery, with life‐saving intervention followed by deferred curative nephrectomy, offering valuable insights into managing such emergencies.

**Abbreviations**
CT: computed tomography
ICI: immune checkpoint inhibitor
IVC: inferior vena cava
MRI: magnetic resonance imaging
PA: pulmonary artery
PE: pulmonary embolism
POD: postoperative day
RA: right atrium
RCC: renal cell carcinoma
TKI: tyrosine kinase inhibitor
VTE: venous thromboembolism

## 1 Introduction

Venous invasion occurs in 4%–10% of RCC cases [1]. Complete resection is the primary treatment; however, tumors extending into the IVC have a poor prognosis, with a 5‐year survival rate of 25%–57% [2]. Tumor thrombus in the RA poses notable challenges [3]. Neves et al. classified IVC thrombus into four levels, known as the Mayo classification: [4] level I (≤ 2 cm from the renal vein), level II (> 2 cm but below the hepatic vein), level III (above the hepatic vein but below the diaphragm), and level IV (above the diaphragm or extending into the RA). Complication rates increase with thrombus level, ranging from 18% to 47% [5]. Single‐stage surgery remains complex, and staged approaches are rare. We present an RCC case with a level IV thrombus and PE, initially treated with life‐saving surgery, followed by curative tumor resection.

## 2 Case Presentation

**FIGURE 1:**
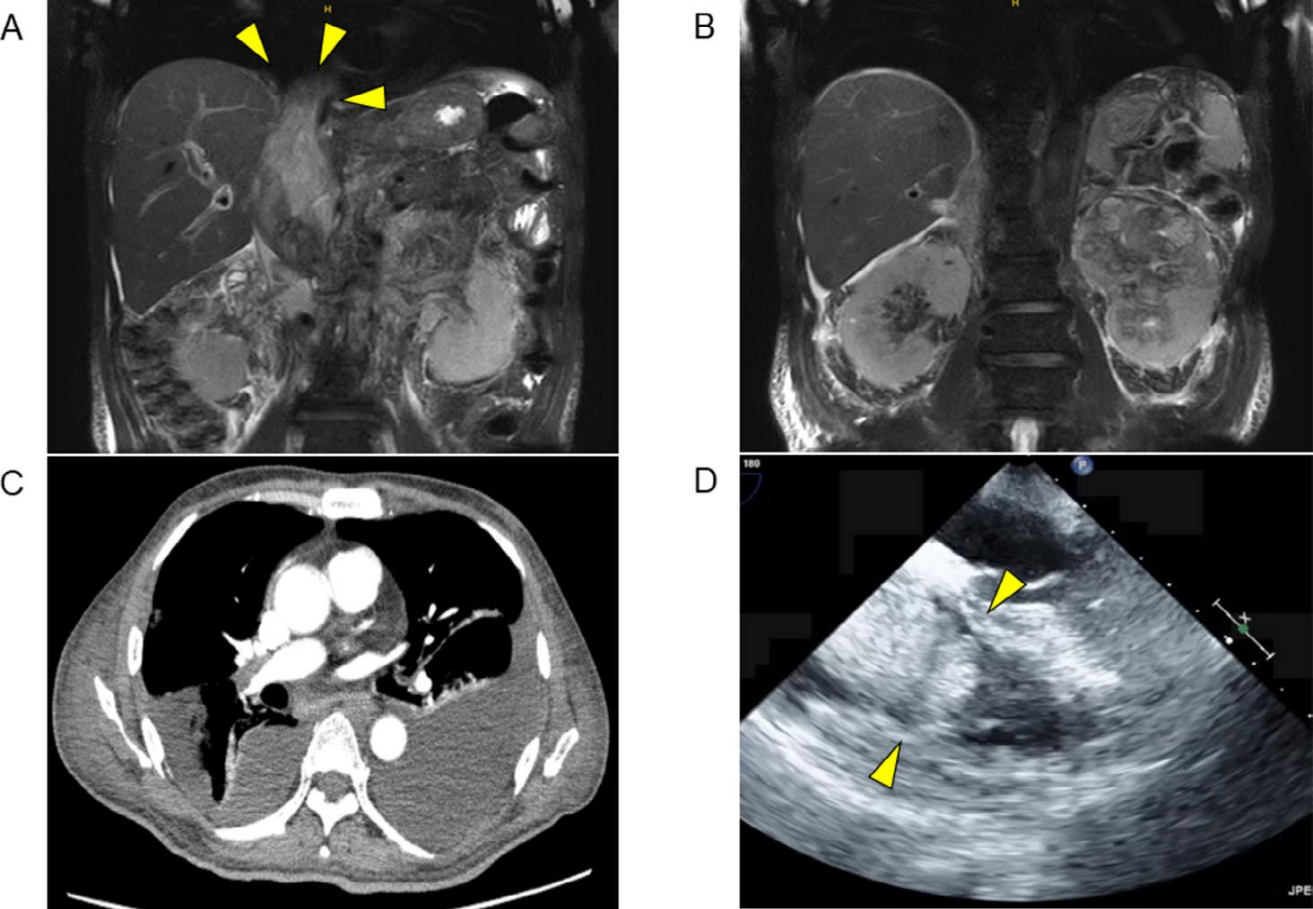
(A, B) T2‐weighted MRI (coronal view). (A) Tumor thrombus in the IVC (arrowhead indicates the cranial end of the IVC tumor). (B) Primary renal tumor. (C) Pulmonary embolism in the right pulmonary artery. (D) Transthoracic echocardiography (arrowhead indicates the tricuspid valve, with the tumor thrombus moving in synchrony with cardiac motion).

**FIGURE 2:**
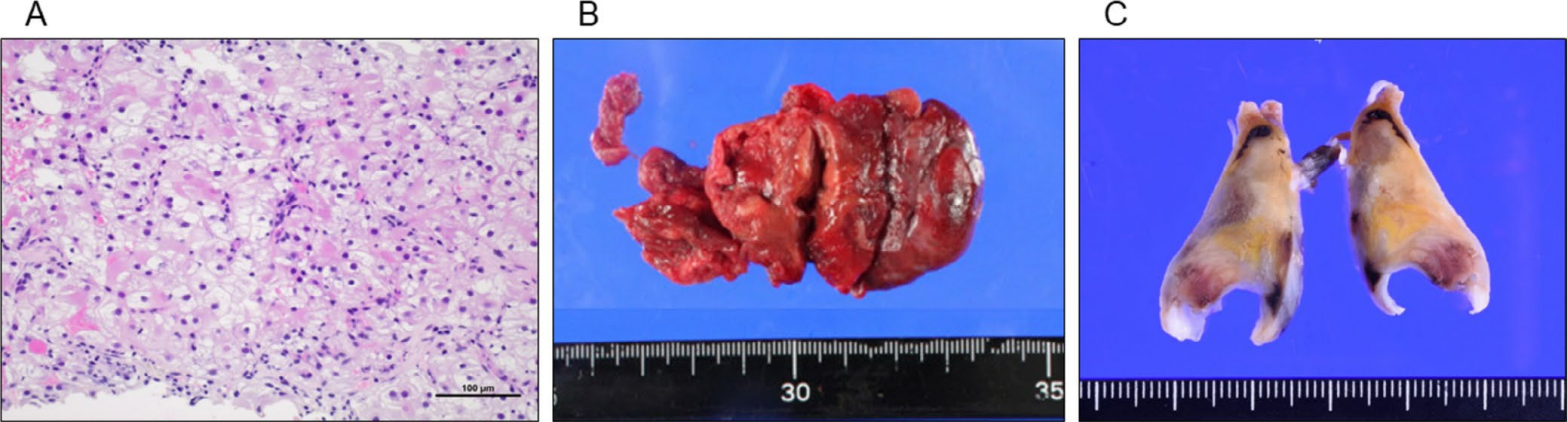
(A) Pathological image of percutaneous renal tumor biopsy, showing clear cell renal cell carcinoma, Fuhrman Grade 3, and WHO/ISUP Grade 3. (B, C) Macroscopic images of the resected tumor thrombus in the right atrium (B) and pulmonary embolism (C); in (C), the yellowish areas correspond to largely necrotic tumor emboli from renal cell carcinoma, while the white to brownish areas represent thrombotic material composed of fibrin and peripheral blood components.

A man in his 60s presented to another hospital with gross hematuria and bilateral leg edema. MRI revealed a left renal tumor and an IVC thrombus extending into the RA (Figure 1A–1B). Biopsy confirmed clear cell RCC (Fuhrman Grade 3, WHO/ISUP Grade 3) (Figure 2A) at stage cT3cN0M0, Mayo level IV. Due to the high‐level extension of the tumor thrombus and suspected adherent bland thrombus, the risk of perioperative complications was deemed substantial, making immediate surgery infeasible. As an approved therapeutic agent, sunitinib therapy was initiated together with anticoagulation to address the suspected thrombotic component.

Two weeks later, sunitinib was discontinued due to Grade 4 thrombocytopenia (4.6 × 10^3^/μL). Axitinib was administered after platelet recovery. Four days later, the patient experienced hypoxemia and chest pain. Contrast‐enhanced CT revealed a PE (Figure 1C), prompting urgent transfer to our hospital.

On arrival, SpO_2_ was 95% on 2 L oxygen. Echocardiography revealed a thrombus in the RA extending through the tricuspid valve (Figure 1D) and signs of right heart strain. To prevent acute heart failure and new PE, emergency embolectomy of the RA and PA was performed via midline sternotomy with cardiopulmonary bypass. The IVC thrombus was partially removed through the RA above the diaphragm. Pathology confirmed RCC with fibrin in the RA thrombus and clots with tumor involvement in the PA thrombus (Figure 2B,C).

**FIGURE 3:**
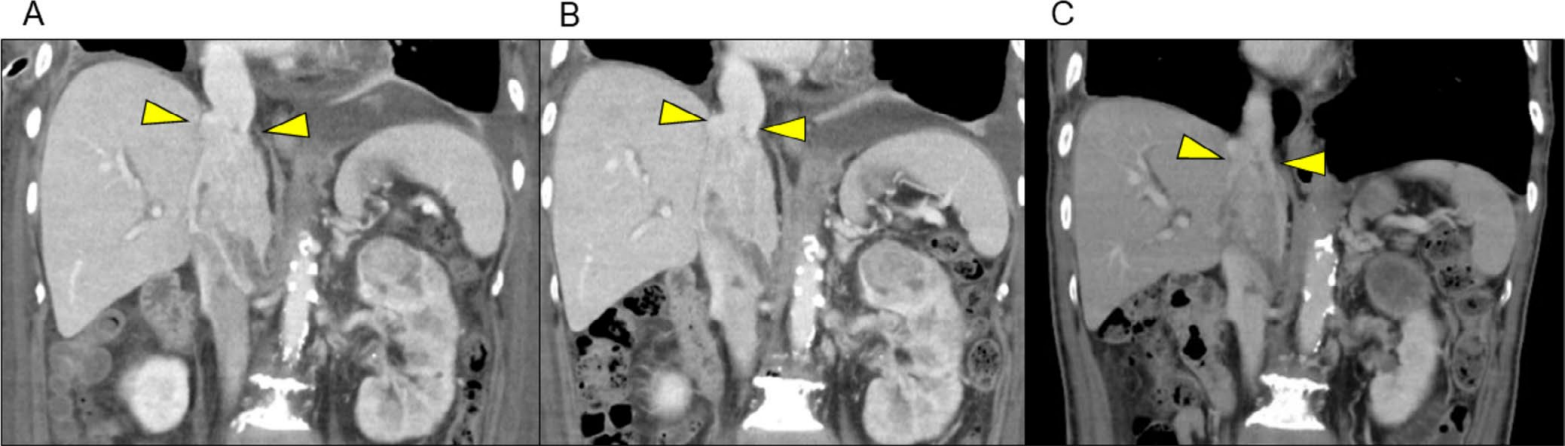
Sequential tumor evaluation following emergency surgery, as shown in coronal CT images. A, B, and C were taken 26, 41, and 67 days postoperatively, respectively. Axitinib was resumed at time (B). Slight reduction of the IVC thrombus and primary renal tumor was observed, with the cranial end of the IVC tumor near the hepatic vein inflow at the time of nephrectomy.

Postoperatively, the patient developed empyema, treated with drainage and antibiotics. Axitinib was resumed on POD 41. After slight shrinkage of the IVC thrombus and primary tumor (Figure 3), with the thrombus just caudal to the hepatic vein inflow (Mayo level III), open radical nephrectomy with IVC thrombectomy and regional lymph node dissection were performed on POD 67. The patient was placed supine, and a Chevron incision was made. After mobilizing the right hepatic lobe to expose the IVC, the tumor thrombus was milked caudally until its cranial margin below the hepatic veins. The IVC was transected above the right renal vein and below the hepatic veins. IVC reconstruction was omitted due to collateral circulation. The hepatoduodenal ligament was pre‐looped for a potential Pringle maneuver, but inflow occlusion was not needed. Pathology showed no IVC wall invasion and lymph node metastasis. Because no residual target lesions remained after surgery and no adjuvant agents were available at that time, observation was chosen.

**FIGURE 4:**
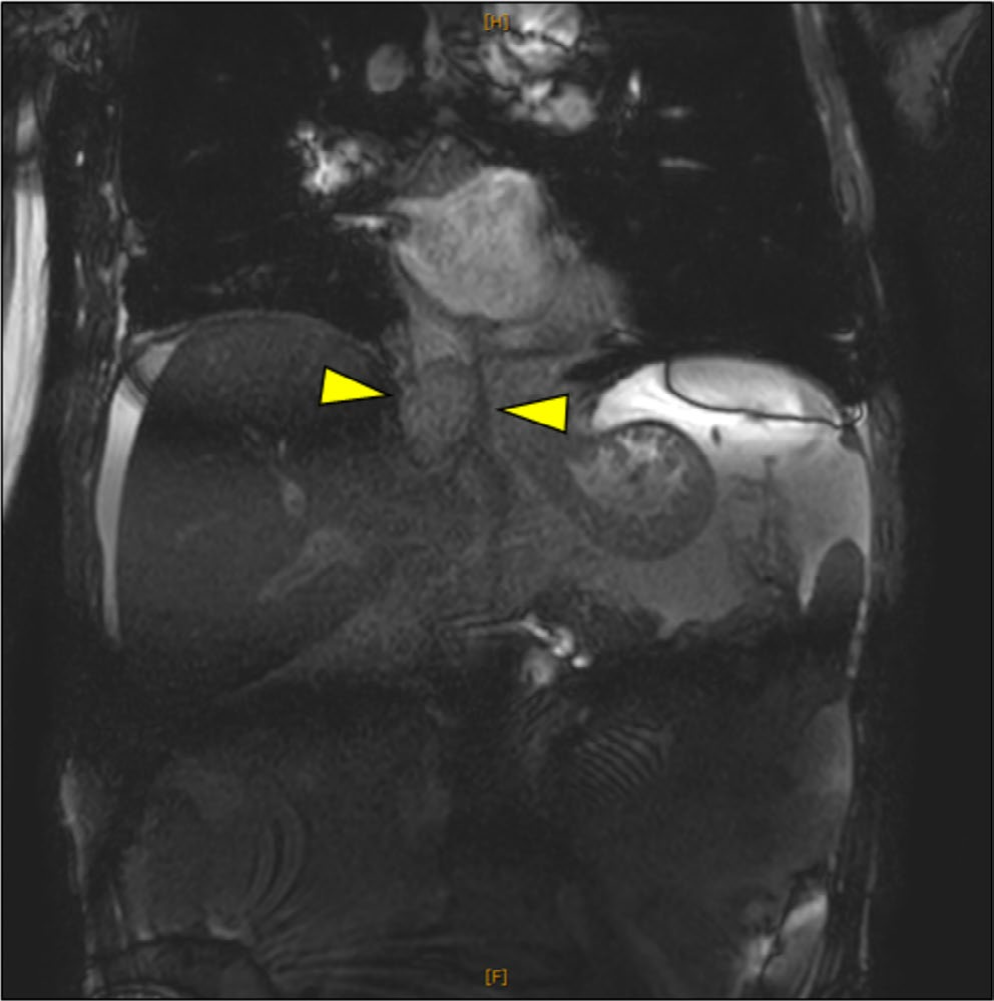
T2‐weighted MRI (coronal view) at the time of recurrence. A mass suggestive of recurrence was identified in the residual IVC, near the hepatic vein inflow.

Postoperatively, the patient developed an abscess at the IVC resection site, treated successfully with antibiotics and drainage. He was discharged 1 month after nephrectomy without recurrence. However, 3 months later, he presented with elevated liver enzymes (AST, 581 U/L; ALT, 620 U/L). MRI revealed a recurrent IVC mass near the hepatic vein (Figure 4). Nivolumab was initiated but was ineffective, and he died of liver failure on POD 130.

## 3 Discussion

In patients with RCC and IVC tumor thrombus, PE does not affect postoperative 90‐day mortality, recurrence, or cancer‐specific mortality if radical nephrectomy is feasible [6]. Few reports describe radical nephrectomy with pulmonary embolectomy, either as a one‐stage [7, 8] or two‐stage [9, 10] procedure, primarily for life‐saving purposes. Although its prognostic effect is unclear, pulmonary embolectomy is justifiable for life‐saving purposes.

Two‐stage removal of level IV tumor thrombus is rare, with one report involving five patients with Budd–Chiari syndrome [11]. The IVC thrombus was excised first, followed by nephrectomy and IVC thrombectomy. A purse‐string suture was placed in the IVC during the first surgery to prevent tumor progression and new PE. Segmental thrombus resection in the RA or IVC may be a reasonable life‐saving strategy, although residual thrombus poses a risk.

Residual thrombus presents a bleeding risk but is generally well‐tolerated within the vascular system. Fragile tumor margins may increase the risk of new thrombotic events like PE. If collateral circulation is sufficient, an IVC purse‐string suture may serve as a preventive measure, as previously reported [11]. Tumor seeding is also a concern but may be acceptable when prioritizing life‐saving surgery.

Postoperatively, the patient developed an abscess at the IVC resection site. Intra‐abdominal abscesses after IVC thrombectomy are uncommon; robotic surgery for Levels II to III thrombus has a 6.3% risk of abscess formation [12]. In our case, no IVC reconstruction was performed, and tumor progression could have filled the remaining IVC, potentially leading to early liver failure. Bypass graft placement requires careful consideration due to infection risk.

RCC patients with venous thrombus have a 6.6‐fold higher risk of VTE than those without [13]. When the thrombus extends above the diaphragm, the risk increases 9.2‐fold compared with thrombus confined to the renal vein and 4‐fold compared with those below the diaphragm. Anticoagulation reduces the VTE risk (HR = 0.56, 95% CI: 0.13–2.48); however, it increases the major bleeding risk (HR = 3.44, 95% CI: 0.95–12.42) [13]. Decisions regarding anticoagulation in RCC with venous thrombus require further study.

Neoadjuvant TKI therapy has resulted in tumor downstaging [14], though no standardized approach exists. Recently, combination therapies of ICIs and ICIs with TKIs have been explored [15, 16]. In the NAXIVA study, axitinib downstaged the Mayo level in 37.5%, with an average tumor reduction of approximately 2 cm [14]. Furthermore, reports of complete response of level IV IVC tumor thrombus with ICI‐based combination therapy have emerged [15]. These findings suggest that, in addition to the tumor‐shrinking effects demonstrated in trials for metastatic RCC—such as the CLEAR trial [17] (71.0% overall response rate for lenvatinib plus pembrolizumab vs. 31.6% for sunitinib) and the CheckMate 214 trial [18] (35% partial response of the primary tumor with nivolumab plus ipilimumab vs. 20% with sunitinib)—ICI‐based combination therapies may also offer significant shrinkage of IVC tumor thrombus.

## 4 Conclusion

Emergency thrombus removal from the PA and RA enabled radical nephrectomy and IVC thrombectomy, demonstrating the feasibility of a staged approach.

## Consent

Informed consent was obtained from the patient, with guarantees of confidentiality.

## Conflicts of Interest

Shusuke Akamatsu is an Editorial Board member of International Journal of Urology and a co‐author of this article. To minimize bias, he was excluded from all editorial decision‐making related to the acceptance of this article for publication.
